# Characterization of ultrasound-mediated delivery of trastuzumab to normal and pathologic spinal cord tissue

**DOI:** 10.1038/s41598-021-83874-x

**Published:** 2021-02-24

**Authors:** Paige Smith, Natalia Ogrodnik, Janani Satkunarajah, Meaghan A. O’Reilly

**Affiliations:** 1 Physical Sciences Platform, Sunnybrook Research Institute, Sunnybrook Health Sciences Centre, 2075 Bayview Avenue, Rm M7-302, Toronto, ON M4N 3M5 Canada; 2 Department of Medical Biophysics, Faculty of Medicine, University of Toronto, Toronto, ON Canada

**Keywords:** Preclinical research, Acoustics

## Abstract

Extensive studies on focused ultrasound (FUS)-mediated drug delivery through the blood–brain barrier have been published, yet little work has been published on FUS-mediated drug delivery through the blood-spinal cord barrier (BSCB). This work aims to quantify the delivery of the monoclonal antibody trastuzumab to rat spinal cord tissue and characterize its distribution within a model of leptomeningeal metastases. 10 healthy Sprague–Dawley rats were treated with FUS + trastuzumab and sacrificed at 2-h or 24-h post-FUS. A human IgG ELISA (Abcam) was used to measure trastuzumab concentration and a 12 ± fivefold increase was seen in treated tissue over control tissue at 2 h versus no increase at 24 h. Three athymic nude rats were inoculated with MDA-MB-231-H2N HER2 + breast cancer cells between the meninges in the thoracic region of the spinal cord and treated with FUS + trastuzumab. Immunohistochemistry was performed to visualize trastuzumab delivery, and semi-quantitative analysis revealed similar or more intense staining in tumor tissue compared to healthy tissue suggesting a comparable or greater concentration of trastuzumab was achieved. FUS can increase the permeability of the BSCB, improving drug delivery to specifically targeted regions of healthy and pathologic tissue in the spinal cord. The achieved concentrations within the healthy tissue are comparable to those reported in the brain.

## Introduction

The blood-spinal cord barrier (BSCB) presents a challenge when treating spinal cord diseases. Similar to the blood–brain barrier (BBB) in both function and morphology, the BSCB limits the passage of large molecule therapeutics (> 500 Da) from the blood stream to the spinal cord parenchyma, preventing the accumulation of these substances in therapeutically relevant concentrations^1,2^. The inability to adequately deliver therapeutics across this barrier has hindered progress in effectively treating a wide range of spinal cord diseases, often leading to poor prognoses upon detection.

Leptomeningeal metastases (LM) occur in 4–15% of patients with solid tumors and the disease has a devastatingly short survival period of only 4–6 weeks when left untreated^3,4^. A combination of radiation to reduce bulky disease and intra-cerebrospinal fluid (CSF) chemotherapy often through a ventricular reservoir to target the whole neuroaxis has been shown to improve survival in some patients (4–8 months), yet there are conflicting reports on whether intra-CSF chemotherapy is effective because of the increased risk of adverse complications during delivery^3^. What is clear is that most systemically delivered drugs have difficulty accumulating in both the tissue and the CSF without administering cytotoxic doses^3^. In patients with human epidermal growth factor receptor 2 positive (HER2 +) metastatic breast cancer, drug therapy with the monoclonal antibody (mAb), trastuzumab, is effective in controlling extra-central nervous system (CNS) metastases but not in controlling the intra-CNS disease^5^. Trastuzumab is a large molecule (~ 148 kDa) that does not readily pass through the healthy BBB or BSCB in large quantities^6^. Although primary and metastatic CNS tumors can lead to an altered, more permeable blood-tumor barrier (BTB), the uptake of systemically delivered trastuzumab to CNS tumors is still quite low compared to other organs and is dependent on the grade and HER2 receptor status of the tumor^6,7^. Therapies which alter the barriers such as radiotherapy can also lead to increases in permeability and thus trastuzumab uptake, yet survival time following radiotherapy remains short^5^.

The application of focused ultrasound (FUS) in the brain in combination with circulating microbubbles has been used with great success in preclinical studies to induce safe, non-ionizing BBB opening^8–10^ and to facilitate the localized delivery of therapeutics^11–14^. This technology is now being used in clinical trials to determine the efficacy of BBB opening and drug delivery in humans^15,16^. The mechanism for BBB opening appears to be multifaceted. It has been shown that changes affecting endothelial cells occur in animals with confirmed BBB opening including vesicle formation, cell fenestration and the widening of the tight junctions between endothelial cells^17^. It is proposed that as FUS interacts with the microbubbles and the microbubbles begin to oscillate, they physically manipulate the proteins at the endothelial cell junctions while expanding and contracting. Additionally, it is suggested that FUS triggers cell fenestration and the formation of vesicles for active transport^17^. Since the BBB and the BSCB are functionally similar, it was proposed that FUS technology could be translated to the spinal cord for safe BSCB opening^18^. A handful of preclinical studies have been published so far showing successful BSCB opening using FUS and additionally, that this technology could facilitate an increased and localized delivery of genes and antibodies^18–23^.

We have previously investigated FUS-induced drug delivery as a means for treating LM in a rat model and a preliminary study showed a potential therapeutic response^21^. It was reported that although there was no statistical difference in survival between the control groups and the FUS + drug treated group, FUS + trastuzumab was effective in reducing the overall tumour burden. Drug delivery to the tumors was not quantified, however, based on prior studies in the brain^24^ it was assumed the reduced tumor burden was the result of enhanced drug delivery mediated by FUS. A similar longitudinal study on FUS-induced trastuzumab delivery to brain metastases found an improvement on survival^14^. Characterizing the drug delivery and distribution to the spinal cord may aid in improving treatment outcomes by facilitating the development of appropriate dosing and treatment schedules.

This study aims to characterize the delivery of trastuzumab to both healthy tissue and tumor deposits within the spinal cord of a rat model of LM following FUS.

## Materials and methods

This study was conceived and executed in compliance with the ARRIVE guidelines for animal research and reporting^25^.

### General animal care procedures

All animal experiments were approved by the Sunnybrook Research Institute animal care committee and were performed in accordance with the guidelines from the Canadian Council on Animal Care. The rats were housed two to a cage prior to surgery or treatment. Rats receiving cell injection catheters were separated following surgery, individually caged, and monitored daily until sacrifice. Cages were equipped with corncob bedding and dry crinkled paper for nesting. Post-surgery, animals were provided with diamond dry cellulose bedding in lieu of corncob bedding (Teklad 7070C, Envio, Indianapolis, IN, USA). Both were changed every 10 days or as needed. Each cage had one PVC tube for shelter and enrichment. The rats had constant access to water and food (Teklad Global 18% Protein Diet, 2918 Pellets; Envigo, Indianapolis, IN, USA). The housing room was temperature controlled between 18° C and 22° C and humidity controlled between 40 and 60%. The lighting was kept on a reverse light cycle, meaning that the room was dark during the day and light at night. Red overhead lights were used when access to the room was needing during the dark period.

### Ultrasound controller

A 580 kHz spherically curved lead zirconate titanate (PZT) transducer (75 mm diameter, F#0.8) was used to deliver the ultrasound. At the fundamental frequency, the lateral beam width is 2.7 mm and the axial beam width is 22.6 mm. Acoustic emissions were monitored passively with a 16 mm, 850 kHz PZT receiver located at the center of the transmit transducer. Both transducers were mounted on an LP-100 treatment system (FUS Instruments, Toronto, ON, Canada) with a three-axis motor-powered positioner. The transducers were submerged in degassed, deionized water. Treatment locations were chosen using MRI guidance at 7 T (BioSpec 70/30 USR; Bruker, Billerica, MA, USA). The transducer was driven at the fundamental frequency in 10 ms bursts at a 1 Hz pulse repetition frequency (PRF) for 2 min. The first 10 pulses at each location were delivered at a low pressure of 0.028 MPa to record the baseline spectral content before bubble activation. Pressure values were based off of calibrations performed in water and were not de-rated for attenuation through tissue. Mean transmission in rat spines at 0.5 MHz has previously been reported as 67 ± 15% (range: 45–92%) and is subject to large local variations due to the bony anatomy of the spine^21^. The signal level to trigger the controller was calculated as the baseline signal plus 10 times the standard deviation of the baseline signal. After the initial 10 pulses, the delivered ultrasound pressure was increased at each location by 0.008 MPa every second until the ultraharmonic signal detected by the receiver reached or surpassed the trigger level, at which point the pressure was reduced by 50%. The pressure level was maintained for the duration of the treatment unless ultraharmonic signal above the trigger level was detected again, leading to another 50% drop in the pressure^26^. The controller can modulate the pressure at each location individually meaning that the pressure drop for each location may be triggered at different times and thus, different peak pressures could be reached.

### General MRI-guided FUS delivery methods

**Figure 1:**
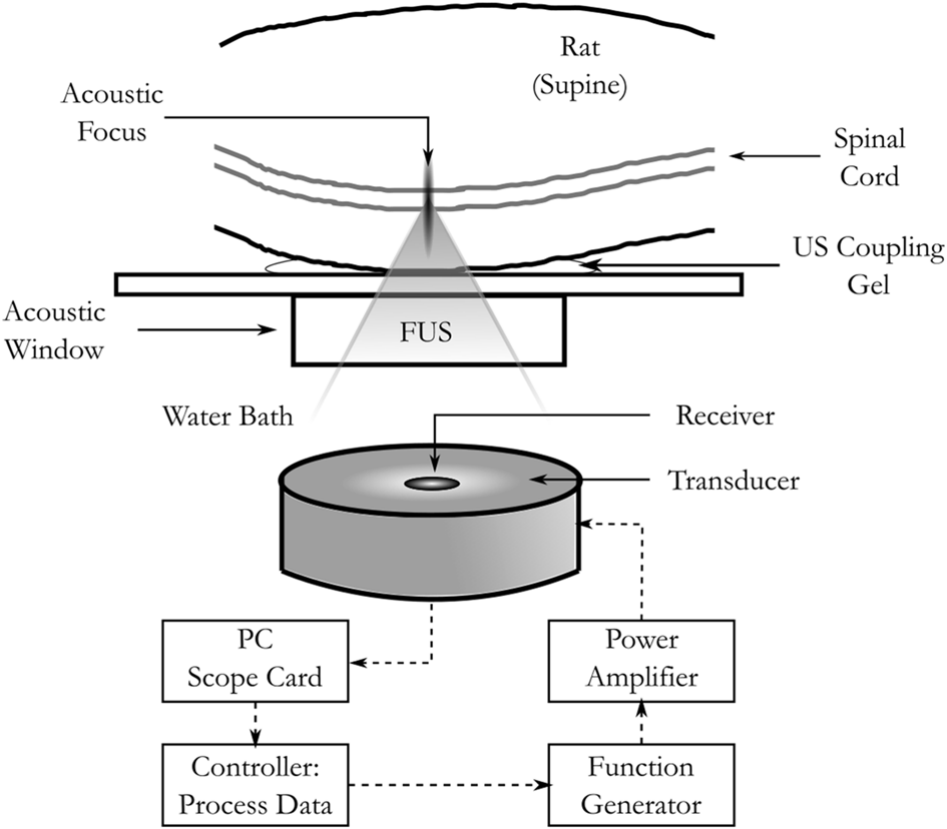
Experimental setup for focused ultrasound delivery to healthy and LM rats.

The FUS treatment system coordinates were registered with the 7 T MRI scanner coordinates at the beginning of each experiment day. Prior to FUS, rats were anesthetized using 2% isoflurane in oxygen while the fur along the back was removed with electric clippers followed by a depilatory cream (Veet; Reckitt Benckiser Group plc, Slough, UK). A 22G or 24G tail vein catheter was inserted to facilitate intravenous injections. All injectables were administered intravenously save the ketamine/xylazine injections which were administered intramuscularly. Rats were placed supine on a sled compatible with both the FUS system and the scanner. Their backs were coupled to an acoustic window using acoustically transparent ultrasound gel and they were switched to 2% isoflurane in sterile air anesthetization for the duration of the treatment. The use of sterile air instead of oxygen during FUS treatments has been shown to prolong the circulation time of microbubbles and improve BBB disruption^27,28^. The experimental setup can be seen in Fig. 1. At the beginning of each treatment, the rats were placed in the scanner and a set of pre-FUS coronal T1w images were acquired to ensure they were positioned within the expected treatment zone.

### Acute drug delivery to healthy spinal cord

**Figure 2:**
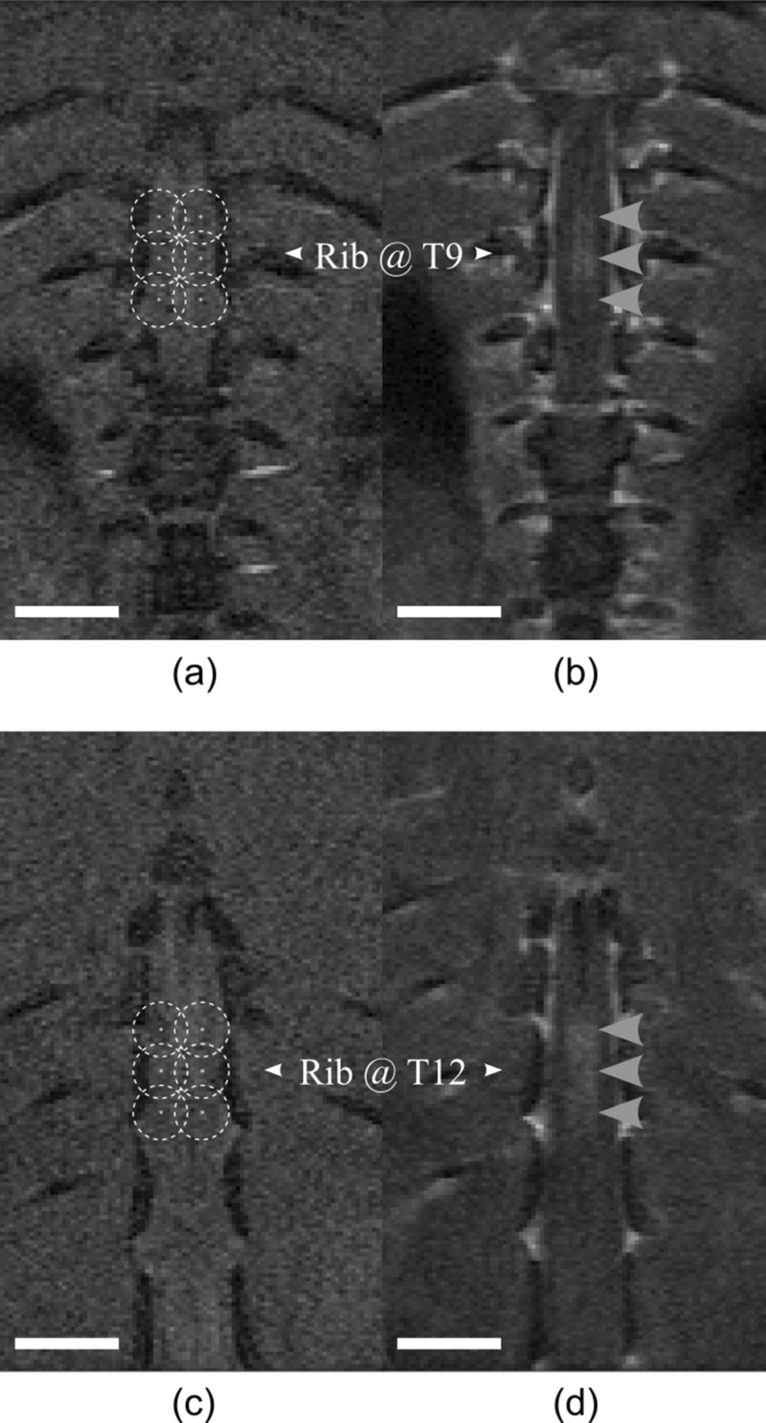
Example T1w coronal MR images pre- and post-FUS for drug delivery to the healthy spinal cord. (**a**, **c**) Pre-FUS treatment planning MR images. Points are spaced at a 2 mm pitch. Dashed circles represent the lateral beam width in water at the fundamental frequency (580 kHz). (**b**, **d**) Post-FUS gadolinium enhanced MR images showing enhancement within the spinal cord following BSCB opening (grey arrowheads). All scale bars = 5 mm.

The distribution of animals in this study is summarized in Table 1. 10 female Sprague–Dawley (SD) rats (220–280 g) were used as a baseline for measuring trastuzumab concentration in normal tissue after combined FUS + mAb treatments. SD rats underwent pre-FUS T1w imaging (FSE, TE = 5.5 ms, TR = 500 ms, 12 averages, RARE factor = 4, FOV = 40 × 40 mm, pixel size = 0.2 × 0.2 mm, slice thickness = 0.5 mm). Due to the limitations of the FUS treatment system, 2 separate sonications were performed with 6 treatment locations per sonication. The 6 treatment spots were arranged in a 3 × 2 grid (2 mm pitch) and centered at thoracic vertebrae 9 (T9) and thoracic vertebrae 12 (T12) (Fig. 2a, c). The first and second sonications alternated between the T9 and T12 location from one animal to the next. The ultrasound for each sonication was delivered as described above. Immediately prior to the first FUS sonication, SD rats received a dose of trastuzumab (8 mg/kg) and then Definity microbubbles (MBs; 0.02 ml/kg). Approximately 5 min later and immediately prior to the second FUS sonication, SD rats received a second dose of Definity MBs (0.02 ml/kg). Gadolinium (0.1 ml/kg; Gadovist, Bayer Inc., Mississauga, ON, Canada) was delivered immediately following the second FUS sonication. Trastuzumab has a long half-life (mean =  ~ 5.8 days at the clinical dose^29^), therefore only one dose was required. The rats were moved from the FUS treatment system to the 7 T scanner. A set of post-FUS coronal T1w images were acquired with identical scan geometry and sequencing as the pre-FUS imaging. The treatment, including BSCB opening, was considered successful if gadolinium enhancement was seen in the region of the spinal cord alongside both T9 and T12. SD rats were divided into two groups. SD group one (N = 5) received Evans blue (50 mg/kg at 2% concentration in saline) immediately after BSCB opening was confirmed and were maintained under anesthesia with ketamine (100 mg/kg) and xylazine (10 mg/kg) for 2 h post-FUS to allow the trastuzumab and Evans blue to extravasate. They were then sacrificed. SD group 2 (N = 5) did not receive Evans blue and were allowed to recover until they were sacrificed 24 h post-FUS. At the point of sacrifice, SD rats were transcardially perfused using ice-cold saline. Two 4 mm treated sections of spinal cord tissue centered at T9 and T12 were removed. Paired untreated control tissue sections of 4 mm in length centered 6 mm above T9 and 6 mm below T12 were also removed. This tissue was flash frozen in liquid nitrogen and stored at − 80 °C.

**Table 1:** Distribution of animals.

Group	Animals (N)	Endpoint (hrs post-FUS)	Assessment
Healthy	5	2	ELISA
Healthy	5	24	ELISA
LM tumors	3	2	Immunohistochemistry

### Characterizing immunoglobulin G (IgG) concentration

Each treated and control section of healthy SD spinal cord tissue was homogenized in a protein extraction buffer (10 µL/1 mg tissue) and aliquoted as necessary to reduce freeze–thaw cycles when using the samples in multiple assays. The protein extraction buffer consisted of the Abcam 10 × RIPA Buffer (10%; ab156034) to perform cell lysis and protein solubilization, the Abcam Protease Inhibitor Cocktail (0.2%; ab65621) to inhibit proteolysis and milliQ water (89.8%). The Thermo matFisher Scientific Pierce BCA Protein Assay Kit (Catalog No. 23225) was used to measure the protein concentration in the tissue lysate samples. The Abcam IgG Human SimpleStep ELISA Kit (Catalog No. ab195215) was used to quantify the concentration of trastuzumab in healthy tissue (measured as total protein) delivered using FUS. The samples were compared against a standard curve of known trastuzumab concentrations in doped tissue. The mean difference between the treated and control tissue sections at each sacrifice timepoint were calculated. The mean difference between the treated sections sacrificed at 2 h versus 24 h was also calculated. The fold change between each treated section and its paired control section was calculated and the mean fold change at 2 h and 24 h post-FUS was compared.

### Cell culture

Human epidermal growth factor receptor 2 positive (HER2 +) MDA-MB-231-H2N cells, established from metastatic mammary adenocarcinoma1^30^, were used to replicate LM. The cells were cultured in 10 cm tissue culture plates in Dulbecco’s Modified Eagle’s Medium (DMEM; Wisent Inc., Montreal, QB, Canada) and 10% fetal bovine serum (FBS; Wisent Inc., Montreal, QB, Canada). The cells were incubated at 37 °C and subcultured at ~ 80% confluency (approximately every 2 days) for 10–12 passages before injection.

### Establishing LM tumor model in RNU rats

Rowett Nude (RNU; lacking T-cells, i.e. immunodeficient) rats (N = 3) were used to model LM. The RNU rats underwent surgery to establish LM following the previously described method by Janczwski et al*.*^31^. In short, the rats were placed under 2% isoflurane anesthesia in oxygen and a laminectomy was performed at L4. A PE-10 catheter was inserted into the subarachnoid space and directed 2 cm cephalad. The free end of the tubing was directed towards the neck, subcutaneously, where it exited at an incision near the lower cervical vertebrae. The rats were allowed to heal for 1 week prior to cell injection. Cultured HER2 + breast cancer cells were resuspended in a 1:1 ratio of media to Matrigel (Corning, Tewksbury, MA, USA) for a concentration of 5 × 10^6^ cells/mL. The suspension was injected via the catheter. Sagittal and axial gadolinium enhanced (gad-) T1w images (FSE, TE = 5.5 ms, TR = 500 ms, 12 averages, rare factor = 4, FOV = 40 × 40 mm, pixel size = 0.2 × 0.2 mm, slice thickness = 0.5 mm), taken on a 7 T preclinical MRI scanner (BioSpec 70/30 USR; Bruker, Billerica MA, USA), were used to confirm that the tumors had seeded and were growing.

### Drug delivery to LM tumor model

**Figure 3:**
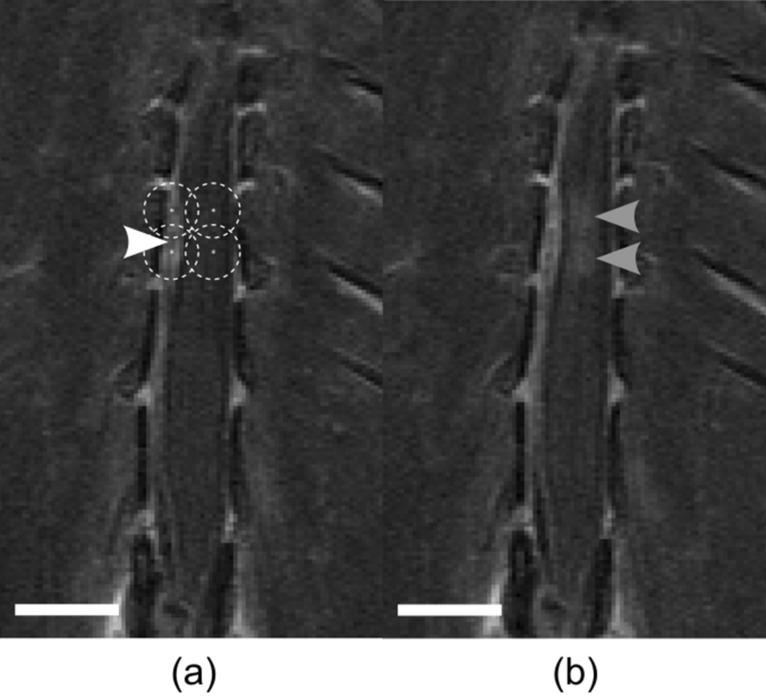
Example gadolinium enhanced T1w coronal MR images pre- and post-FUS for drug delivery to a rat model of LM. (**a**) Pre-FUS treatment planning MR image. Points are spaced at a 2 mm pitch. The leftmost points fall within the tumor boundary (white arrowhead) and the rightmost points fall adjacent to serve as a positive control for BSCB opening. Dashed circles represent the lateral beam width in water at the fundamental frequency (580 kHz). (**b**) Post-FUS MR image showing enhancement within the spinal cord following BSCB opening (grey arrowheads). All scale bars = 5 mm.

Tumors were first visible on MRI in the RNU rat spinal cords 6 days post-cell injection. The rats were treated with FUS at 12 days post-cell injection. They received a pre-treatment dose of the MR contrast agent gadolinium (0.1 ml/kg) and a set of pre-FUS coronal T1w images were acquired to precisely locate the tumors. The leaky vasculature of tumors allows for the extravasation of gadolinium into the otherwise non-enhancing spinal cord tissue. Using these images, four treatment locations in a 2 × 2 grid (2 mm pitch) were selected such that 2 locations were within the tumour boundary and 2 locations were adjacent to the tumor boundary, within the healthy tissue (Fig. 3a). Immediately prior to the FUS sonication, RNU rats received a dose of gadolinium (0.1 ml/kg), trastuzumab (8 mg/kg), and then Definity microbubbles (0.02 ml/kg; Lantheus Medical Imaging, Billerica, MA, USA). The ultrasound was delivered as described above. Following FUS exposure, the rats were moved from the FUS system to the 7 T scanner. A set of post-FUS coronal T1w images were acquired with identical scan geometry and sequencing as the pre-FUS imaging. The treatment was considered successful if gadolinium enhancement was seen at the sonication locations both within and outside of the tumour boundaries. RNU rats were then removed from the scanner and received Evans blue (50 mg/kg at 2% concentration in saline) and maintained under anesthesia with ketamine (100 mg/kg) and xylazine (10 mg/kg) until they were sacrificed later at 2 h post-FUS. At this point, the RNU rats were transcardially perfused using saline followed by 10% neutral buffered formalin. A 50 mm section of the spinal cords were excised starting at L4 towards the head, then the dura mater was removed. Photos of the spinal cords alongside a ruler were taken to locate the treated region marked by extravasated Evans blue. The formalin fixed RNU rat spinal cords were sliced into 5 μm thick coronal sections at 100 μm intervals. Coronal sections were chosen to cut perpendicular to the long axis of the FUS and so the slices would be in agreement with the coronal MR images taken. The tissue was stained with Hematoxylin and Eosin (H&E) to visualize cell morphology and red blood cell extravasations. Immunohistochemistry was performed on adjacent sections using an Abcam recombinant anti-human IgG antibody (Catalogue No. ab109489) to visualize the delivery of trastuzumab.

### Image analysis

MRI data were analyzed using the Medical Image Processing, Analysis, and Visualization (MIPAV) software^32^ to measure post-FUS gadolinium enhancement, estimate BSCB opening volume, and estimate tumor volumes. Hyperintense regions within the spinal cord on each slice of the post-FUS gadolinium enhanced T1w images were traced to determine the enhanced area. The areas were multiplied by the slice thickness of 0.5 mm and summed to produce an estimation of the opening volume. Gadolinium enhancement was assessed as a percent change. This was done by averaging the signal in a 3 × 3 voxel region of interest around the peak intensity voxel for each target location and comparing it to the average of a 3 × 3 voxel region outside of the target location.

All histology slides were digitized using a Zeiss AxioImager microscope (Zeiss, Berlin, Germany). A K-nearest neighbors (KNN) supervised clustering algorithm was designed and implemented in MATLAB (MathWorks, Natick, MA, USA) to semi-automatically segment the digitized H&E images into binary masks of tumor and healthy tissue and to measure the tumor volumes. The FIJI^33^ distribution of ImageJ^34^ was used to perform semi-quantitative analysis of trastuzumab concentration in the LM rats from the digitized slides. The built-in colour deconvolution function for DAB staining was used to separate the colour channel associated with the anti-human IgG antibody. Targeted and untargeted regions of both healthy spinal cord tissue and tumor tissue were contoured and the mean grey-level value was measured. Similar to the methods proposed in Nguyen et al.^35^, grey-level values were subtracted from the maximum value of 255 resulting in a reciprocal intensity to present a direct relationship between the measured value and increased trastuzumab concentration. To account for variability between animals, intensity values were compared as ratios with their untreated counterparts e.g., treated grey matter/untreated grey matter. The healthy tissue values were considered the baseline since these concentrations were directly measured with an ELISA in the healthy rats who were treated with similar exposures. The ratio between treated tumor tissue and treated healthy tissue was also calculated. The healthy tissue was assumed to be an average of the grey and white matter values to mimic the homogenization of the tissues during the ELISAs. Due to the fact that DAB staining is non stoichiometric, this method cannot be used as a direct nor absolute quantification of trastuzumab concentration in the tissue of the LM rats.

### Statistical analysis

Significant differences in concentration between 2 groups was determined using a two-tailed *t* test following an f-test for equal variance with a 95% confidence interval. The linear correlation between the percent change in gadolinium enhancement and total trastuzumab concentration was assessed by determining the Pearson coefficient (R). The Pearson coefficient was converted to a t-statistic to calculate a two-tailed t-distribution and asses the *p* value of the Pearson coefficient (95% confidence interval).

## Results

BSCB opening (Fig. 2b, d) was seen following FUS in all SD rats except one from the 2-h sacrifice group. This rat was the first one treated on the day of the experiment and after adjusting the system calibration parameters and retreating, opening was seen. It was possible that the incorrect calibration meant that the first treatment had missed the spinal cord entirely so this rat was given Evans blue to be further assessed at the time of sacrifice. The average acoustic peak negative pressure (PNP) reached during SD treatments was 0.36 ± 0.09 MPa. The rats sacrificed 2 h-post FUS received Evans blue dye which was also visible at the treated locations in all SD spinal cords following sacrifice and excision. The rat that was treated twice had more disperse Evans blue dye outside of the treated locations so we speculate that mild off-target BSCB opening may have been achieved following the first treatment. Thus, this animal was excluded from further analysis. The average BSCB opening volume across all healthy rats was 6.4 ± 2 mm^3^ with an average increase in enhancement of 67 ± 30%.

**Figure 4:**
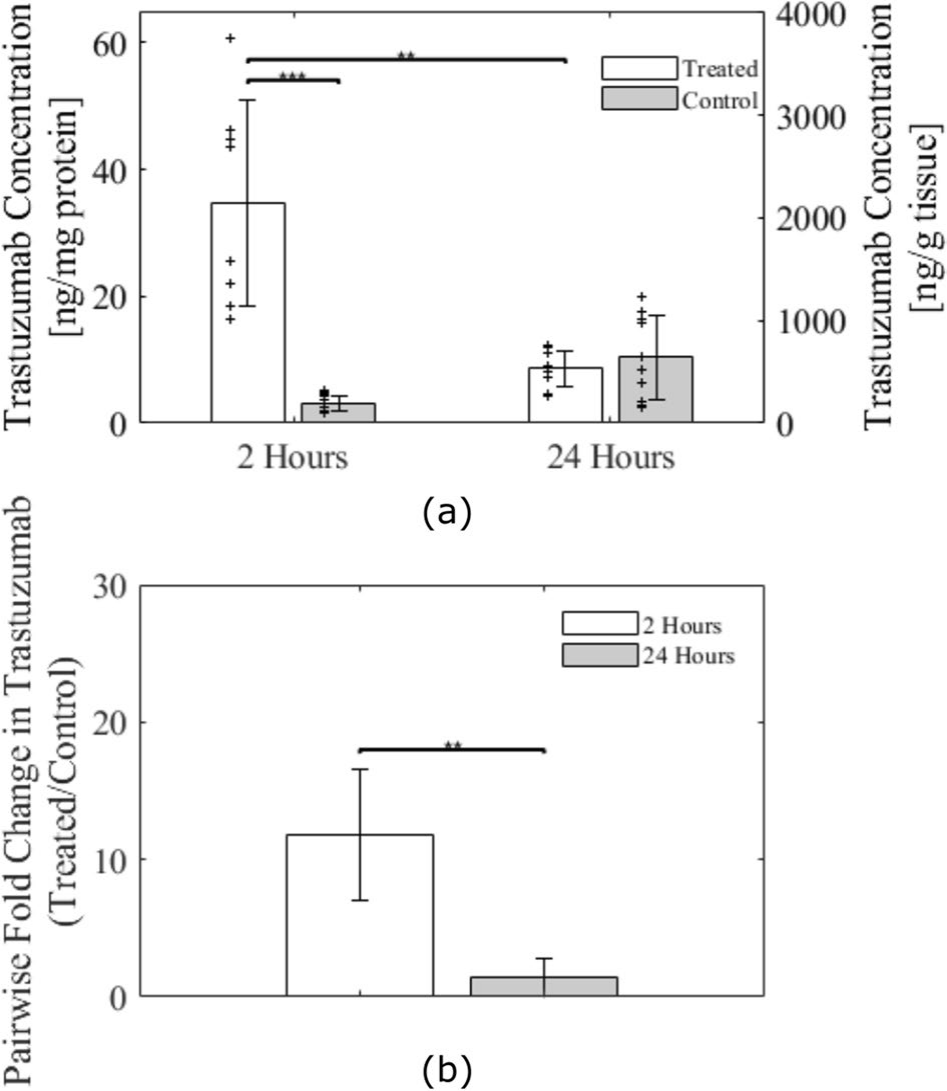
ELISA results for trastuzumab concentration in healthy rat spinal cord tissue. (**a**) Comparison between FUS-targeted regions and untargeted regions at 2-h post-FUS and 24-h post-FUS. Individual data plotted as ‘ +’ scatter points. (**b**) Fold increase in trastuzumab concentrations from untargeted to FUS-targeted region at 2-h and 24-h post-FUS. Significance was assessed on a 95% confidence interval. Error bars show standard deviation.

**Figure 5:**
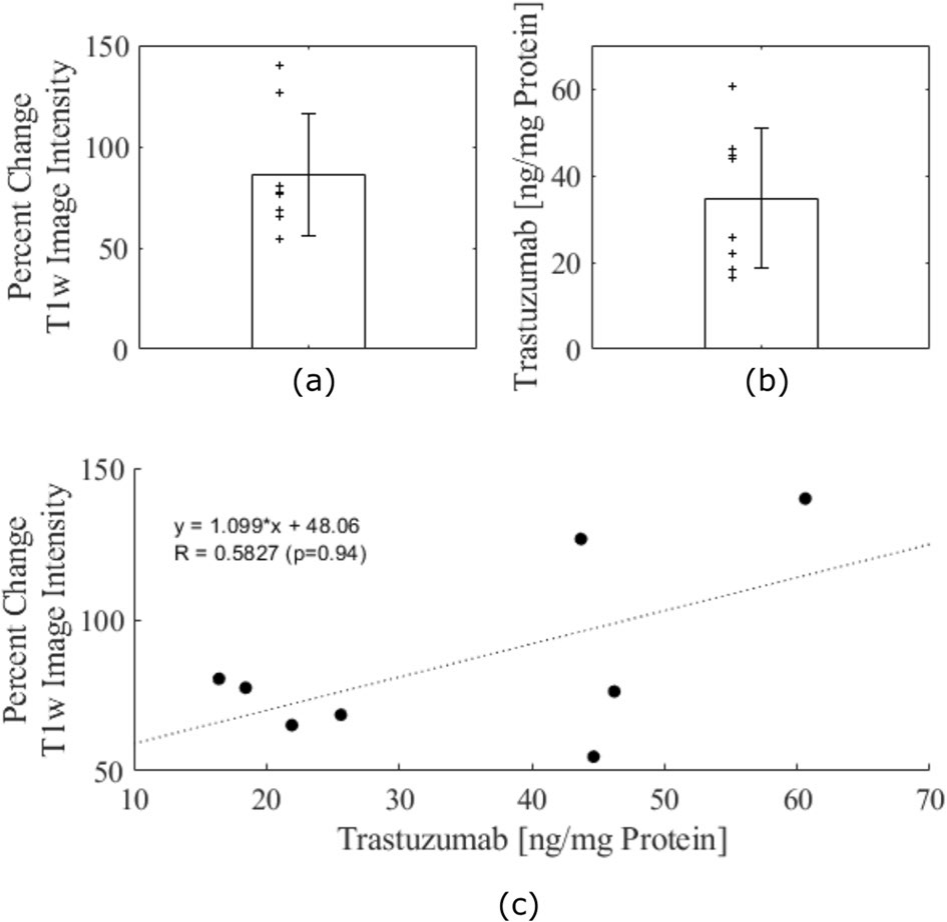
Comparison in healthy rats sacrificed 2 h post-FUS of the percent change in MR enhancement in post-FUS gadolinium enhanced T1w images and trastuzumab concentration. (**a**) Bar graph of average percent change in T1w image intensity following FUS in rats sacrificed at 2-h post-FUS. Individual data plotted as ‘ + ’ scatter points. (**b**) Bar graph of average trastuzumab concentration in ng/mg at 2-h post-FUS. Individual data plotted as ‘ + ’ scatter points. (**c**) Linear regression of trastuzumab concentration and percent change in T1w MR image intensity. Poor correlation is seen (R = 0.5827; *p* = 0.94). Error bars show standard deviation.

The ELISA was highly sensitive to trastuzumab with a minimum detectable dose of 0.05 ng/mL. The amount of trastuzumab detected was significantly higher at 2-h post-FUS than at 24-h post-FUS (Fig. 4a). Between treated and untreated control samples, there was a 12 ± fivefold increase in trastuzumab at 2-h (Fig. 4b). There was no statistical difference between treated and untreated samples at 24-h (1.4 ± 1.4-fold increase). Linear regression yielded an R value of 0.58 (*p* = 0.94), showing that there is a poor correlation between the ELISA measured trastuzumab concentrations at 2-h and the gadolinium enhancement following FUS (Fig. 5c). The average increase in MR enhancement in the 2-h sacrifice group was 86 ± 30% (Fig. 5a) and the average concentration of trastuzumab in the tissue at 2-h post-FUS was 35 ± 16 ng/mg of protein (Fig. 5b).

In the LM tumor group, BSCB opening was achieved in all 3 rats, evidenced by hyperintense regions within the tumors and healthy tissue at the tumor periphery on gadolinium enhanced T1w images (Fig. 3b). The average peak acoustic pressure reached during RNU treatments was 0.46 ± 0.09 MPa PNP. Evans blue dye extravasation was also visible on all RNU spinal cords following sacrifice and excision.

**Figure 6:**
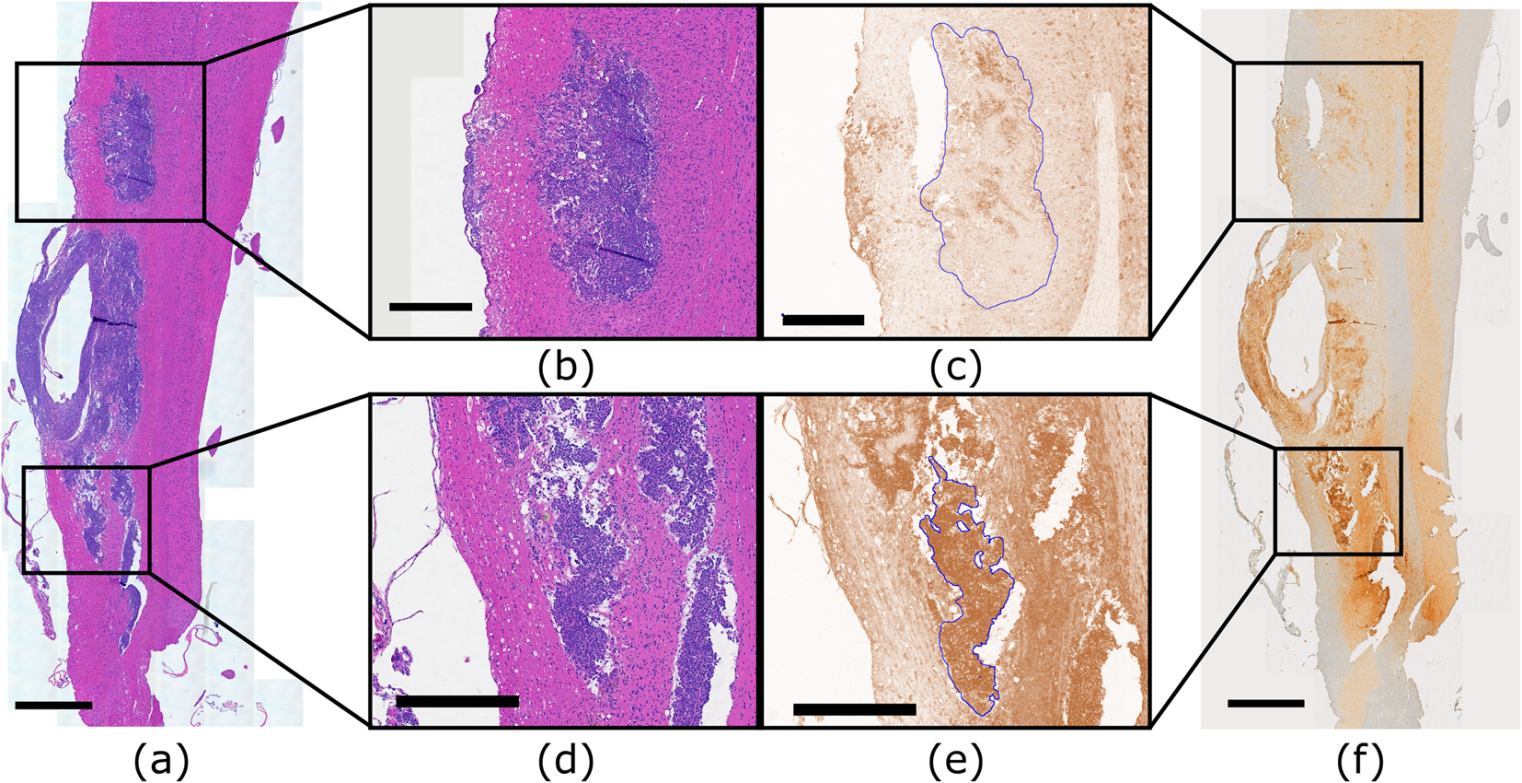
Spinal cord tissue of an LM rat prepared for histology showing tumor burden and trastuzumab distribution. (**a**, **b**, **d**) Stained with hematoxylin and eosin to view gross tumor morphology. (**c**, **e**) Stained with an anti-human IgG antibody to detect trastuzumab distributed throughout the tissue then deconvolved in ImageJ to visualize DAB signal. (**b**, **c**) Enlarged views of untargeted tumor tissue. ROI contoured in blue in slide c. (**d**, **e**) Enlarged views of targted tumor tissue. ROI contoured in blue in slide e. (**f**) Stained with an anti-human IgG antibody to detect trastuzumab distributed throughout the tissue. Scale bars: (**a**, **f**) 1 mm; (**b**–**e**) 0.5 mm.

**Figure 7:**
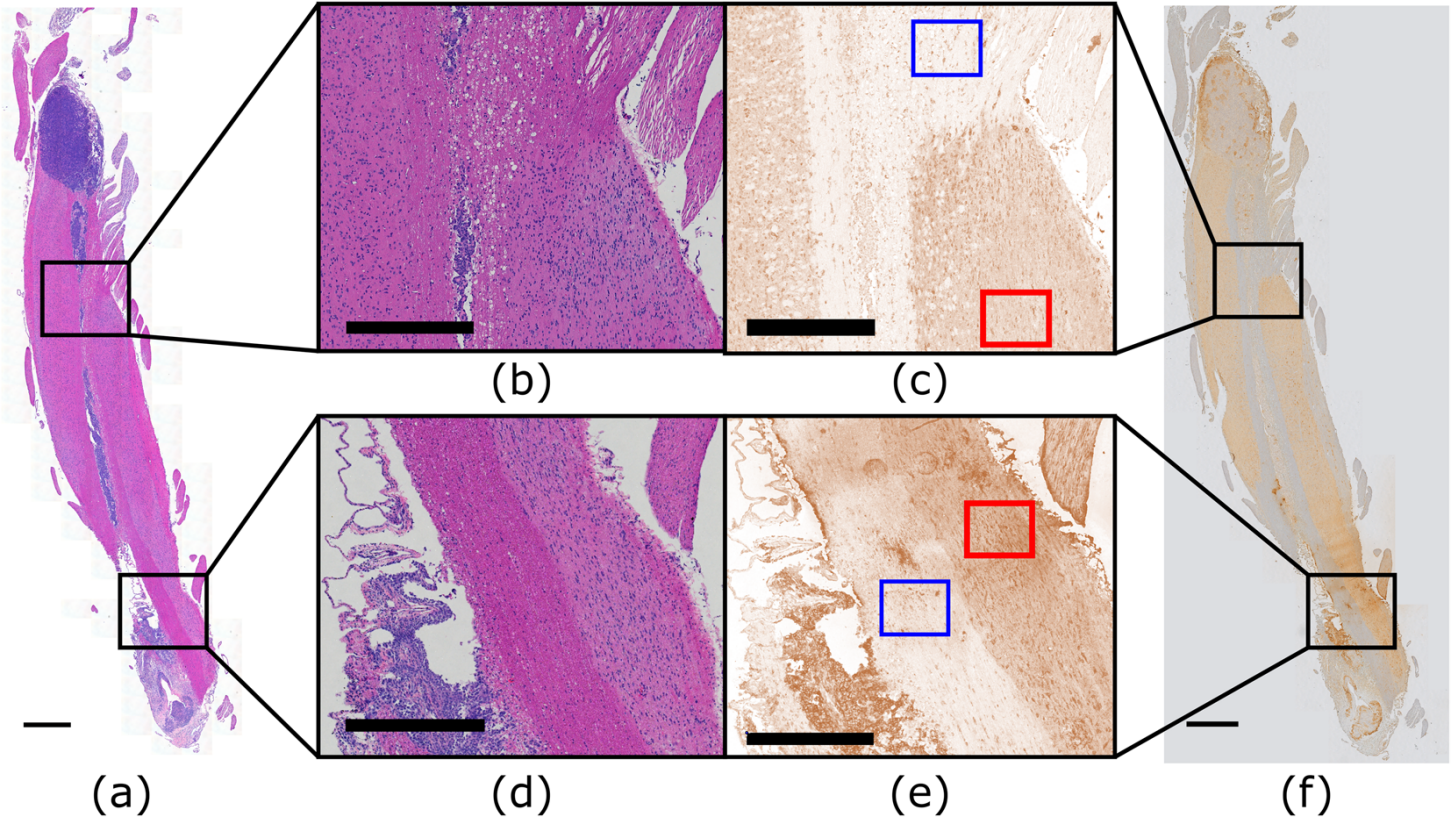
Spinal cord tissue of an LM rat prepared for histology showing tumor burden and trastuzumab distribution. (**a**, **b**, **d**) Stained with hematoxylin and eosin to view gross tumor morphology. (**c**, **e**) Stained with an anti-human IgG antibody to detect trastuzumab distributed throughout the tissue then deconvolved in ImageJ to visualize DAB signal. (**b**, **c**) Enlarged views of untargeted healthy tissue. White matter ROI contoured in blue and grey matter ROI contoured in red in slide c. (**d**, **e**) Enlarged views of targeted healthy tissue. White matter ROI contoured in blue and grey matter ROI contoured in red in slide e. (**f**) Stained with an anti-human IgG antibody to detect trastuzumab distributed throughout the tissue. Scale bars: (**a**, **f**) 1 mm; (**b**–**e**) 0.5 mm.

**Figure 8:**
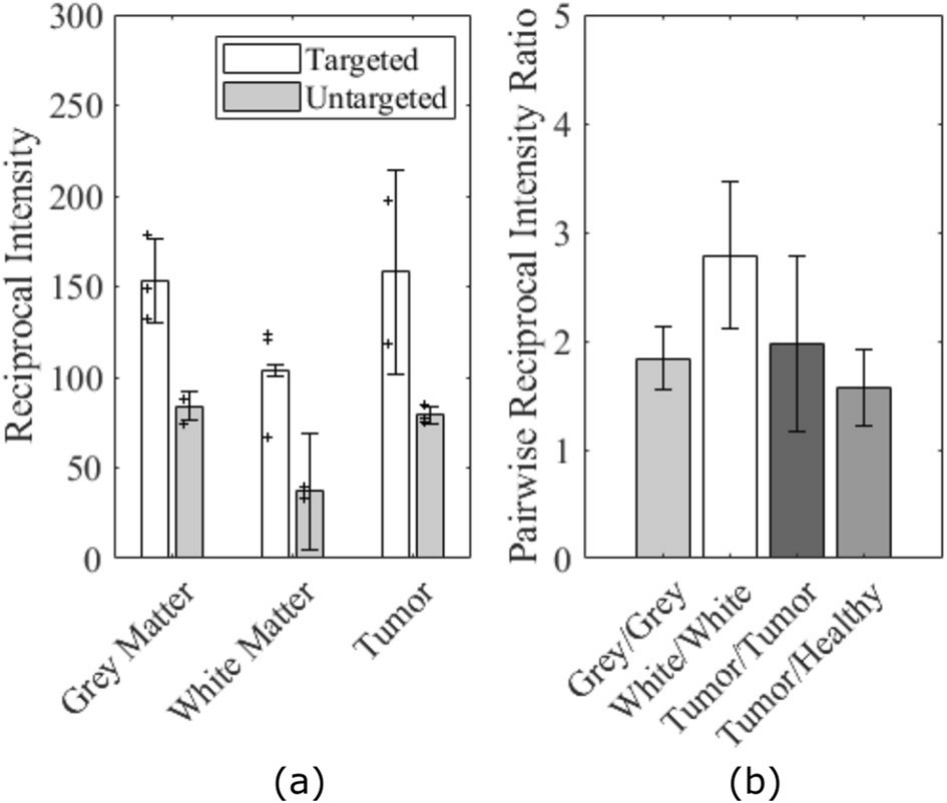
Reciprocal intensity analysis results in LM tumor rats. (**a**) Comparison between FUS targeted and untargeted regions in different tissue types. (**b**) Ratio between paired targeted and untargeted reciprocal intensity values in different tissue types and the ratio between targeted tumor and targeted healthy tissue reciprocal intensities. Error bars show standard deviation.

The KNN segmentation algorithm segmented the H&E slides into background, healthy spinal cord and tumor tissue. From this segmentation, the tumors had an average volume of 4 ± 3 mm^3^. Representative images of the tumor presentation can be seen in Figs. 6a and 7a. Qualitative analysis of the anti-IgG stained slides revealed that different tissue structures expressed different staining levels both within and outside of the FUS targeted regions, exemplified in Figs. 6f and 7f. This was supported by the semi-quantitative analysis of reciprocal intensity values performed in ImageJ. Figure 6b–e show examples of targeted and untargeted tumor tissue ROIs and Fig. 7b–e show examples of targeted and untargeted healthy tissue ROIs in both the grey and white matter. The tumor and white matter ROIs are outlined in blue (Figs. 6c, e, 7c, e) and the grey matter ROIs are outline in red (Fig. 7c, e). The ROIs are all presented on the deconvolved DAB signal images that were used to produce the reciprocal intensity measurements. The treated grey matter, white matter, and tumor, all had higher reciprocal intensities than the untargeted tissue structures (Fig. 8a). After pairwise matching, the mean ratio of reciprocal intensity values was highest for the white matter at a value of 2.8 ± 0.7 (Fig. 8b). The grey matter and tumor ratios were closer at 1.8 ± 0.3 and 2.0 ± 0.8, respectively. The ratio of targeted tumor to targeted healthy reciprocal intensity was 1.6 ± 0.4.

## Discussion

Our results show that FUS with microbubbles can significantly increase the concentration of the large molecule therapeutic trastuzumab within the healthy spinal cord and LM deposits by temporarily increasing BSCB permeability. At 2 h post-FUS we note a trastuzumab concentration of 35 ng/mg protein or 2191 ng/g tissue. The achieved concentration in the tissue is comparable with previous reports in the brain^24^, however, our histological findings illustrate that unlike that previous study we did not observe hemorrhage or red blood cell extravasations, which we attribute to the use of actively controlled treatment exposures.

Trastuzumab was present within the sonicated regions of the healthy spinal cord at statistically significant concentrations 2 h following FUS, but there was no statistical difference between sonicated and unsonicated healthy tissue by 24 h following FUS. This suggests the availability of unbound trastuzumab in the spinal cord parenchyma is less than 24 h. Since the ELISA measurements were only performed on healthy rat tissue samples, this clearance timeline may not be representative of the rats with tumors. In healthy rats, the concentration measurements are primarily of free antibody that has not bound to a HER2 receptor. HER2 + breast cancer cells, like the ones used for this model of LM, overexpress HER2 and can have up to 2 million receptors so trastuzumab would more readily bind^36^.

The literature has limited information on the time course and mechanism of clearance of trastuzumab from the CNS. However, IgG is a monoclonal antibody of similar size and is reported to have a rapid efflux across the BBB after intra-cerebral injection, with a half-life of ~ 48 min^37^. The neonatal Fc-receptor (FcRn) plays a role in efflux of IgG variants across the BBB^38,39^. While the effects of FUS on FcRn have not been studied, suppression of the important BBB efflux transporter P-glycoprotein has been observed following FUS^40^, the magnitude of the change is influenced by the exposure conditions and degree of BBB opening. Conversely, others have reported that FUS effects are insufficient to overcome transporter-mediated efflux of the dual ABCB1/ABCG2 substrate erlotinib^41^. The impact of FUS on receptor-mediated trafficking across the BBB and BSCB is a complex subject that merits additional consideration in future work. However, based on our knowledge of the time-course of clearance of IgG, the observation of clearance of unbound trastuzumab within 24hrs is not unreasonable.

Trastuzumab has a long half life, reported as mean 5.8 days (range: 1–32 days) on the product insert and as high as 28 days in published studies, and serum concentrations remain high for several days following injection. However, the blood–brain barrier has been reported to close by 24 h and as early as 6 h post-FUS^42–44^. It is possible that repeated FUS-induced BSCB opening hours or days after the initial injection of trastuzumab could restore higher than baseline levels of free antibody to the spinal cord parenchyma without repeated injections, but this would have to be investigated further. In the brain it has been shown that a second sonication at a later timepoint, without re-administering the drug being delivered, can enhance extravasation^45,46^.

Direct quantification via ELISA of the trastuzumab concentration in the tumor tissue of the LM model was not performed because of the challenge of isolating the thin tumor tissue from the rest of the spinal cord. As the FUS beam width is approximately the width of the whole spinal cord it is expected that trastuzumab will be delivered to the healthy tissue adjacent to the targeted tumor tissue. Failure to isolate the tumor tissue from the adjacent healthy tissue would confound the concentration measurements leading to inaccurate results. However, accurate measurements of trastuzumab concentration in the tumor tissue as well as the time course of clearance may be beneficial for planning repeat longitudinal treatments in future studies.

We showed that in healthy subjects the increased antibody concentration following FUS is localized within the sonicated region. On histology, qualitatively we see that non-sonicated tumors have some heterogenous staining attributed to leaky tumor vasculature, but the staining within the sonicated tumor masses was more homogeneous. The reciprocal intensity measurements were higher for the sonicated tumors suggesting that a higher concentration of trastuzumab was delivered to these regions. These results are consistent with FUS studies in brain tumors showing enhanced delivery across the blood-tumor barrier^46–49^. Although white matter showed lower absolute staining it had the largest ratio of reciprocal intensity after FUS. White matter is less vascularized than grey matter so a lower background signal is expected. The reciprocal intensity ratio of 1.6 ± 0.4 between targeted tumors and healthy tissue suggests that FUS is facilitating at least equal or higher delivery of trastuzumab to the sonicated tumors as was measured by ELISA in the healthy group. The reciprocal intensity ratios in the healthy tissue cannot be compared directly with the pairwise ELISA measurements at 2-h post-FUS since the grey level values are bound between 0 and 255 but, both showed a large increase in the value of the targeted regions against the untargeted regions of the same spinal cord which act as an internal control.

The lack of correlation between trastuzumab concentration and gadolinium enhancement is contrary to the results shown by Kinoshita et al*.*^24^ in the brain and may be due to the different FUS parameters used. The study in the brain used fixed pressures of 0.6 or 0.8 MPa FUS in 10 ms bursts at a 1 Hz PRF for 40 s and also fit data through their control animals where FUS was not applied. In the present study we used actively controlled exposures and did not include control data in the fit, resulting in clustering of the data and no trend observed. In the work by Kinoshita, the barrier integrity also seems to have been compromised more extensively in the brain treatments, evidenced by severe red blood cell extravasation and TUNEL positive staining in some treated locations. No red blood cell extravasations were observed in the present work in the acute tumor study and these rats were treated in the same manner as the healthy rats. More intense BBB opening would lead to more intense gadolinium extravasation and subsequent tissue enhancement. At the lower treatment pressures reached in the spine sonications, gadolinium extravasation was always achieved but it was more variable. Individual circulation dynamics may also contribute to the variability, although the gadolinium dose was scaled by body weight to minimize this effect.

The correlation between MRI contrast and tissue accumulation of agents varies in the literature. Some studies have reported correlations between dyes^50,51^ or therapeutics, such as reported by Kinoshita. Others have reported no correlation. Dynamic contrast enhanced (DCE) MRI has been used as a quantitative imaging technique to measure BBB permeability by calculating the transfer constant (K^trans^) of gadolinium from the blood plasma to the extravascular and extracellular space^52–56^. This calculation considers the rate at which gadolinium accumulates in the plasma, known as the arterial input function, to produce an accurate estimate of K^trans^. Although more robust than a comparison of percent enhancement, DCE-MRI may not be suitable for direct measurement of trastuzumab accumulation. Park et al*.*^54^, found that DCE-MRI had good correlation with the accumulation of the small molecule chemotherapy agent doxorubicin in the brain. However, work from the same group found no correlation between DCE-MRI and concentration of the larger liposomal doxorubicin^46^, illustrating that differences in molecule size and pharmacokinetics make estimates of drug concentrations in the tissue based on small molecule MRI tracers challenging. Further, given the thin sheet-like structure of the leptomeningeal metastases, achieving sufficient SNR to get accurate DCE-MRI measurements within the tumor tissue, in a rodent model, would be challenging.

The controller used in this study has been used by our group previously in many studies for opening the BBB^57–59^. Focusing ultrasound into the spinal canal presents different challenges than focusing sound into the skull cavity. The issue of target motion due to breathing is reduced by placing the animals supine so that the chest can expand freely however, the posterior elements of the vertebral bone are highly irregular especially in comparison to the skull surface. In rats, an average of about a 33% signal loss is expected from bone attenuation, although with large spatial variations^21^, but large focal distortions are not expected since the vertebrae are thin. It was expected that using the same controller for this study as used in the small animal BBB studies would provide similar opening. Translating FUS induced BSCB opening to the clinic involves minimizing focal distortions and eliminating standing waves within the relatively small cavity of the spinal canal. A handful of recent studies have looked to address this with promising results^19,60–62^, including application in a large animal model^20^.

## Conclusions

FUS in combination with microbubbles was able to safely and temporarily increase the permeability of the blood-spinal cord barrier to increase the concentration of the monoclonal antibody trastuzumab in the cord parenchyma (35 ± 16 ng/mg of protein at 2 h) to similar concentrations as achieved in the brain. The increased concentration was localized to the treatment targets and was seen in both healthy tissue and tumor deposits within a rat model of LM. This increase returns to baseline in healthy tissue by 24 h. Reciprocal intensity analysis of the anti-human IgG staining suggests that the concentration of trastuzumab in the tumor tissue is equal to or greater than that in the healthy tissue. More work on the optimal dosing scheme and additional delivery routes, such as intrathecally, will need to be done to advance this treatment clinically.

## Data Availability

Original data are available from the authors upon request.

## References

[1] Bartanusz V, Jezova D, Alajajian B, Digicaylioglu M (2011). The blood-spinal cord barrier: Morphology and clinical implications. Ann. Neurol..

[2] Pardridge WM (2005). The blood-brain barrier: Bottleneck in brain drug development. NeuroRx.

[3] Chamberlain MC (2010). Leptomeningeal metastasis. Curr. Opin. Oncol..

[4] Taillibert S, Chamberlain MC (2018). Leptomeningeal metastasis. Handb. Clin. Neurol..

[5] Pieńkowski T, Zielinski CC (2009). Trastuzumab treatment in patients with breast cancer and metastatic CNS disease. Ann. Oncol..

[6] Terrell-Hall TB, Nounou MI, El-Amrawy F, Griffith JIG, Lockman PR (2017). Trastuzumab distribution in an in-vivo and in-vitro model of brain metastases of breast cancer. Oncotarget.

[7] Yonemori K (2010). Disruption of the blood brain barrier by brain metastases of triple-negative and basal-type breast cancer but not HER2/neu-positive breast cancer. Cancer.

[8] Choi JJ, Pernot M, Small SA, Konofagou EE (2007). Noninvasive, transcranial and localized opening of the blood-brain barrier using focused ultrasound in mice. Ultrasound Med. Biol..

[9] Hynynen K, McDannold N, Vykhodtseva N, Jolesz FA (2001). Noninvasive MR imaging–guided focal opening of the blood-brain barrier in rabbits. Radiology.

[10] Nhan T (2013). Drug delivery to the brain by focused ultrasound induced blood-brain barrier disruption: Quantitative evaluation of enhanced permeability of cerebral vasculature using two-photon microscopy. J. Control. Release.

[11] Choi JJ, Wang S, Tung YS, Morrison B, Konofagou EE (2010). Molecules of various pharmacologically-relevant sizes can cross the ultrasound-induced blood-brain barrier opening in vivo. Ultrasound Med. Biol..

[12] Jordão JF (2010). Antibodies targeted to the brain with image-guided focused ultrasound reduces amyloid-β plaque load in the TgCRND8 mouse model of Alzheimer’s disease. PLoS ONE.

[13] Lin CY (2016). Non-invasive, neuron-specific gene therapy by focused ultrasound-induced blood-brain barrier opening in Parkinson’s disease mouse model. J. Control Release.

[14] Park E, Zhang Y, Vykhodtseva N, Mcdannold N (2012). Ultrasound-mediated blood–brain/blood-tumor barrier disruption improves outcomes with trastuzumab in a breast cancer brain metastasis model. J. Control Release.

[15] Carpentier A (2016). Clinical trial of blood-brain barrier disruption by pulsed ultrasound. Sci. Transl. Med..

[16] Mainprize T (2019). Blood-brain barrier opening in primary brain tumors with non-invasive MR-guided focused ultrasound: A clinical safety and feasibility study. Sci. Rep..

[17] Sheikov N, McDannold N, Vykhodtseva N, Jolesz F, Hynynen K (2004). Cellular mechanisms of the blood-brain barrier opening induced by ultrasound in presence of microbubbles. Ultrasound Med. Biol..

[18] Wachsmuth J, Chopr R, Hynynen K (2009). Feasibility of transient image-guided blood-spinal cord barrier disruption. AIP Conf. Proc..

[19] Fletcher SMP, Ogrodnik N, O’Reilly MA (2020). Enhanced detection of bubble emissions through the intact spine for monitoring ultrasound-mediated blood-spinal cord barrier opening. IEEE Trans. Biomed. Eng..

[20] Fletcher SMP, Choi M, Ogrodnik N, O’Reilly MA (2020). A porcine model of transvertebral ultrasound and microbubble-mediated blood-spinal cord barrier opening. Theranostics.

[21] O’Reilly MA (2018). Preliminary investigation of focused ultrasound-facilitated drug delivery for the treatment of leptomeningeal metastases. Sci. Rep..

[22] Payne AH (2017). Magnetic resonance imaging-guided focused ultrasound to increase localized blood-spinal cord barrier permeability. Neural Regen. Res..

[23] Weber-Adrian D (2015). Gene delivery to the spinal cord using MRI-guided focused ultrasound. Gene Ther..

[24] Kinoshita M, McDannold N, Jolesz FA, Hynynen K (2006). Noninvasive localized delivery of Herceptin to the mouse brain by MRI-guided focused ultrasound-induced blood-brain barrier disruption. Proc. Natl. Acad. Sci..

[25] Kilkenny C, Browne W, Cuthill IC, Emerson M, Altman DG (2010). Animal research: Reporting in vivo experiments: The ARRIVE guidelines. Br. J. Pharmacol..

[26] O’Reilly MA, Hynynen K (2012). Blood-brain barrier: Real-time feedback-controlled focused ultrasound disruption by using an acoustic emissions–based controller. Radiology.

[27] Mullin L (2011). Effect of anesthesia carrier gas on in vivo circulation times of ultrasound microbubble contrast agents in rats. Contrast Media Mol. Imaging.

[28] McDannold N, Zhang Y, Vykhodtseva N (2017). The effects of oxygen on ultrasound-induced blood-brain barrier disruption in mice. Ultrasound Med. Biol..

[29] U. S. Food and Drug Administration. Herceptin. https://www.accessdata.fda.gov/drugsatfda_docs/label/1998/trasgen092598lb.pdf (1998).

[30] Du Manoir JM (2006). Strategies for delaying or treating In vivo acquired resistance to trastuzumab in human breast cancer xenografts. Clin. Cancer Res..

[31] Janczewski KH (1998). A simple, reproducible technique for establishing leptomeningeal tumors in nude rats. J. Neurosci. Methods.

[32] McAuliffe MJ (2001). Medical image processing, analysis and visualization in clinical research. Proc. IEEE Symp. Comput. Med. Syst..

[33] Schindelin J (2012). Fiji: An open-source platform for biological-image analysis. Nat. Methods.

[34] Schneider CA, Rasband WS, Eliceiri KW (2012). NIH Image to ImageJ: 25 years of image analysis. Nat. Methods.

[35] Nguyen D, Zhou T, Shu J, Mao J-H (2013). Quantifying chromogen intensity in immunohistochemistry via reciprocal intensity. Cancer InCytes.

[36] Iqbal N, Iqbal N (2014). Human epidermal growth factor receptor 2 (HER2) in cancers: Overexpression and therapeutic implications. Mol. Biol. Int..

[37] Zhang Y, Pardridge WM (2001). Mediated efflux of IgG molecules from brain to blood across the blood-brain barrier. J. Neuroimmunol..

[38] Cooper PR (2013). Efflux of monoclonal antibodies from rat brain by neonatal Fc receptor, FcRn. Brain Res..

[39] Challa DK, Velmurugan R, Ober RJ, Ward ES (2014). FcRn: From molecular interactions to regulation of IgG pharmacokinetics and functions. Curr. Top Microbiol. Immunol..

[40] Aryal M (2017). Effects on P-glycoprotein expression after blood-brain barrier disruption using focused ultrasound and microbubbles. PLoS ONE.

[41] Goutal S (2018). Physical blood-brain barrier disruption induced by focused ultrasound does not overcome the transporter-mediated efflux of erlotinib. J. Control Release.

[42] Boekhout AH, Beijnen JH, Schellens JHM (2011). Trastuzumab. Oncologist.

[43] Leyland-Jones B (2003). Pharmacokinetics, safety, and efficacy of trastuzumab administered every three weeks in combination with paclitaxel. J. Clin. Oncol..

[44] O’Reilly MA, Hough O, Hynynen K (2017). Blood-brain barrier closure time after controlled ultrasound-induced opening is independent of opening volume. J. Ultrasound Med..

[45] Yang FY, Lin YS, Kang KH, Chao TK (2011). Reversible blood-brain barrier disruption by repeated transcranial focused ultrasound allows enhanced extravasation. J. Control Release.

[46] Aryal M, Park J, Vykhodtseva N, Zhang YZ, McDannold N (2015). Enhancement in blood-tumor barrier permeability and delivery of liposomal doxorubicin using focused ultrasound and microbubbles: Evaluation during tumor progression in a rat glioma model. Phys. Med. Biol..

[47] Park J, Aryal M, Vykhodtseva N, Zhang YZ, McDannold N (2017). Evaluation of permeability, doxorubicin delivery, and drug retention in a rat brain tumor model after ultrasound-induced blood-tumor barrier disruption. J. Control Release.

[48] Sun T (2017). Closed-loop control of targeted ultrasound drug delivery across the blood–brain/tumor barriers in a rat glioma model. Proc. Natl. Acad. Sci..

[49] Zhang Z, Xue Y, Liu Y, Shang X (2010). Additive effect of low-frequency ultrasound and endothelial monocyte-activating polypeptide II on blood-tumor barrier in rats with brain glioma. Neurosci. Lett..

[50] Choi HS (2015). Permeability parameters measured with dynamic contrast-enhanced MRI: Correlation with the extravasation of evans blue in a rat model of transient cerebral ischemia. Korean J. Radiol..

[51] Chu, P. C. *et al.* Pharmacodynamic analysis of magnetic resonance imaging-monitored focused ultrasound-induced blood-brain barrier opening for drug delivery to brain tumors. *Biomed Res. Int.* **2013**, (2013). 10.1155/2013/627496 PMC3626247 23607093

[52] Tofts PS, Kermode AG (1991). Measurement of the blood-brain barrier permeability and leakage space using dynamic MR imaging. 1. Fundamental concepts. Magn. Reson. Med..

[53] Vlachos F, Tung YS, Konofagou EE (2010). Permeability assessment of the focused ultrasound-induced blood-brain barrier opening using dynamic contrast-enhanced MRI. Phys. Med. Biol..

[54] Park J, Zhang Y, Vykhodtseva N, Jolesz FA, McDannold NJ (2012). The kinetics of blood brain barrier permeability and targeted doxorubicin delivery into brain induced by focused ultrasound. J. Control Release.

[55] Chai WY (2014). Magnetic-resonance imaging for kinetic analysis of permeability changes during focused ultrasound-induced blood-brain barrier opening and brain drug delivery. J. Control Release.

[56] McMahon D, Oakden W, Hynynen K (2020). Investigating the effects of dexamethasone on blood-brain barrier permeability and inflammatory response following focused ultrasound and microbubble exposure. Theranostics.

[57] Alkins R (2013). Focused ultrasound delivers targeted immune cells to metastatic brain tumors. Cancer Res..

[58] Burgess A (2014). Alzheimer disease in a mouse model: Mr imaging-guided focused ultrasound targeted to the hippocampus opens the blood-brain barrier and improves pathologic abnormalities and behavior. Radiology.

[59] Mcmahon D, Bendayan R, Hynynen K (2017). Acute effects of focused ultrasound-induced increases in blood-brain barrier permeability on rat microvascular transcriptome. Sci. Rep..

[60] Fletcher SP, Reilly MAO (2018). Analysis of multifrequency and phase keying strategies for focusing ultrasound to the human vertebral canal. IEEE Trans. Ultrason. Ferroelectr. Freq. Control.

[61] Xu R, O’Reilly MA (2018). Simulating transvertebral ultrasound propagation with a multi-layered ray acoustics model. Phys. Med. Biol..

[62] Xu R, O’Reilly MA (2019). A spine-specific phased array for transvertebral ultrasound therapy: Design and simulation. IEEE Trans. Biomed. Eng..

